# Revisiting the doctor’s role at the primary healthcare clinic

**DOI:** 10.4102/safp.v62i1.5242

**Published:** 2020-11-06

**Authors:** Bernhard Gaede

**Affiliations:** 1 Department of Family Medicine, Faculty of Health Sciences, University of KwaZulu-Natal, Durban, South Africa

**Keywords:** primary healthcare, PHC, individual patients, healthier community, doctor

## Abstract

With the growing evidence regarding the benefit of a primary healthcare (PHC) approach to both individual patients and for a healthier community, a number of policy initiatives in South Africa are aimed at strengthening services at subdistrict level. Historically, the role of the doctor in many PHC clinics in South Africa had been limited to a clinical role. However, in the context of wanting to have a greater impact on social determinants of health, the role of the doctor at the PHC clinic needs to be revisited. A wider role of the doctor, in the context of an expanded multidisciplinary team is being explored.

## Introduction

The roles and functions of doctors (and all categories of healthcare professionals) performing within the healthcare system vary greatly and have been shaped by history and traditions, resource availability and both national and local policies that shape the healthcare system. In strengthening the primary healthcare (PHC) system in South Africa, an important area of policy development is to re-examine the role of doctors in the system, particularly the greater involvement and impact that they can have on the health of individuals and communities.

The PHC approach, starting with the Declaration of Alma Ata in 1978, is strongly advocated internationally^1^ and continues to shape many healthcare systems throughout the world. Doctors have been a central resource in all healthcare systems yet their role in PHC varies considerably, from doctor-driven general practices to family health teams in Brazil or community medicine approaches in India.

### Development of primary healthcare in South Africa

A major contributor to how PHC has been conceptualised internationally has its roots in South Africa: community oriented primary care (COPC).^2,3^ In the seminal work of Sidney and Emily Kark in Pholela in the midlands of now-KwaZulu-Natal, the systematic engagement with the communities sought to address some of the social determinants of illness. The doctor’s role stretched beyond dealing with the medical condition of patients and connected clinical care with community-level impact. The doctor played a central role in an expanded team that involved community members, healthcare professional and team members from sectors outside of the health services.

In the district healthcare system that developed post-1994, the PHC system is a largely nurse-run service and had a strong focus on the PHC clinic.^4^ The doctor’s visits to the clinic has been a common feature and particularly in rural areas where the local district hospital was supporting the PHC clinics in its catchment area. Such a geographic link is critical in the District Health System (DHS) where each level of care is responsible for the population of their drainage area, rather than merely the sick patients who present themselves are admitted to the ward.^5^ Doctors’ visits to the clinics were to some degree dependent on the local staffing levels and established routines, with many clinics being visited once or twice a week – but varying from no visits at all to having a number of doctors permanently based at the larger clinic (or Community Health Centre).^6^ The roles of the visiting doctor also varied considerably and in some instances included training and quality improvement, but with most of them doing only clinical work.^7,8^ But even within this, depending on the location of the practice (urban vs. rural, hospital vs. clinic vs. ‘subdistrict’), the range of procedural skills being performed varied greatly.

## The current South African policy context: Re-engineering primary healthcare

In 2013, a health system reform of re-engineering PHC in South Africa was introduced that was initially modelled on approaches in Cuba and Brazil^9^ and conceptually drawing on the COPC approach.^10^ It conceptualised the service in a subdistrict and its existing services and added a number of streams including ward-based outreach teams, school health services, a district level specialist outreach team and contracting of doctors to support the PHC clinics.^10,11,12^ It implied a range of functions that would work together coherently to provide services to the defined population of the subdistrict. The logic of the concept is to explicitly link the service provision with addressing social determinants of health.^9,11^

### Implementing the policy

At the time, most of the work that doctors were doing was to review chronic care prescriptions and seeing a few referrals from the nurses. The expanded role of doctors’ at the clinic visits can draw on previous explorations.^7,8,10^ In the literature on outreach, simple outreach is considered to comprise of only providing clinical services whereas complex outreach involves a number of activities such as clinical governance, mentoring and training. The Latter has been found to be more effective than merely a displaced outpatient service.^13,14^

In order to understand the role of the doctor at the clinic, we need to evaluate the current models of care in relation to how they respond appropriately to the needs of individuals, families or households and communities. In the move towards a more person- and people-centered care.^15,16^ The World Health Organisation also explored how the different levels can function in much greater unity^17^ and emphpasised the ‘false dichotomy’ of hospital versus PHC – and that in the way the hospital services engage, can have a profound transformative effect on developing holistic and people-centred care.

## Exploring new roles

How can we think of the role of the doctor at the clinic and what does it look like? What role do doctors have in preventative medicine and health promotion? Do doctors participate in rehabilitation of patients? And what about palliative care?

Consider this hypothetical situation:

Mrs. Dlamini, a 58-year-old widow was discharged from hospital following a below-knee amputation of her right leg for a ‘diabetic foot’. She had been taking medication for type 2 diabetes mellitus for a few years before this admission and had been followed up by the local Community Health Worker (CHW) at home. The doctor at the local clinic reviewed her every 6 months and prior to developing the diabetic foot, the doctor repeatedly expressed concerned that her control was ‘borderline’ and had spoken to her about adherence to medication, exercise and diet – particularly to cut down on the amount of sugar and fat in her diet. She had been referred to a dietician and also been given pamphlets on healthy living.

While she was in hospital, her glucose levels were much better and she was seen by the dietician, physiotherapist and the occupational therapist. She mobilized well after the amputation and learnt to use crutches to get around, and slowly gained confidence. She hoped for a prosthesis and was given follow-up appointments at the hospital with the various team members.

After being discharged the CHW was very glad to see her back home and visited her regularly. The CCG motivated her to continue to mobilize and they discussed the hope for a prosthesis. The family was very concerned and supportive and tried and assist where they could. The terrain at home made it difficult to go far, but she tried to remain active. The wound was healing slowly and still required some dressing changes with which her younger daughters assisted.

After a week at home, Mrs. Dlamini noticed a bad smell coming from the wound that looked more inflamed. The CHW urged to get to clinic, which had become more difficult due to mobility and she was delayed for a few days until she managed to arrange transport from a distant nephew. When she reached the clinic, the sister indicates that the wound had become septic and that her ‘sugar has gone high’ again.

A scenario such as this may resonate for many clinicians. Everyone seemed to be trying to do their best and provide the best care – but somehow Mrs. Dlamini ended up with a septic wound on the amputated leg. Where were the gaps in the provision of care? Were not all the key team members of the multidisciplinary team (MDT) involved and fulfilled their professional roles?

Yet, clearly, something was missing: the intention of the individual tasks somehow was not aligned with an overall view of Mrs. Dlamini and on that level, the approach lacked cohesion and coordination. For example, the MDT had little information from the CHW regarding the situations at home in terms of diet, environment or resources to support her, the doctors at the hospital did not know where Mrs. Dlamini lived, how she was going to go home or cope after the amputation at home. What would realistic targets for her glycaemic control be in the context of her home circumstances?

## Possibilities of different focus: Community oriented primary care cycle

Onreflection, I would like to explore how the visiting doctor potentially can impact, but needs to understand how an impact can be made in Mrs. Dlamini’s life, in the context of how the system works. Having an ecological world view – seeing the person in the context of her home, the community and population creates a context of the consultation and forms the basis of intervention. The COPC approach offers a structured system of how the consultation can link into the wider context.^18^

**FIGURE 1:**
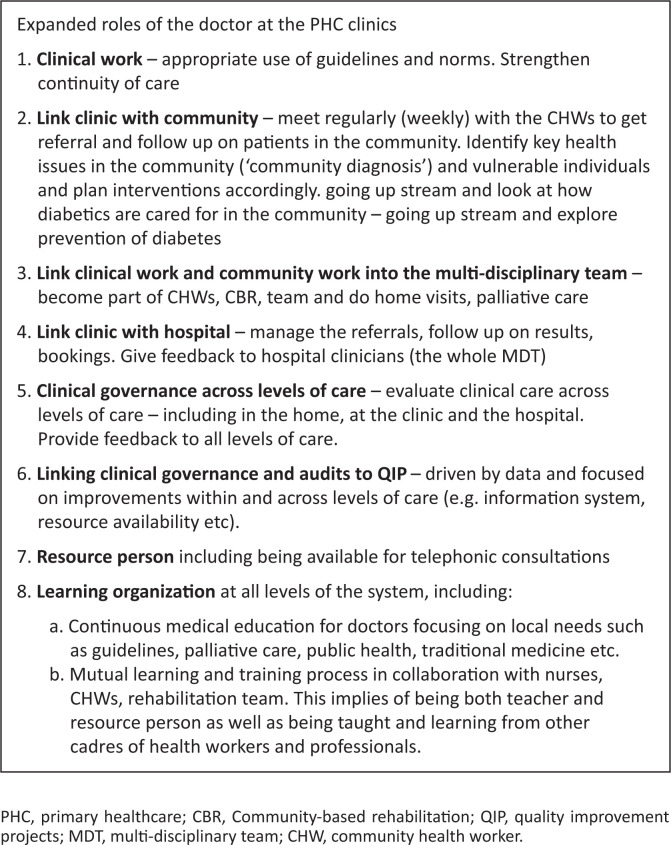
Expanded roles of the doctor at the primary healthcare clinic.

Through a process of being able to track the information about Mrs. Dlamini from home to the hospital and back, a more coherent and supportive approach can be crafted. It would link CCG’s to decisions in the clinics and the hospital, link the MDT to care of amputees in the community, link the doctors and therapists at the hospital to follow-up at clinic and community.^10,12^ Some of the solutions in this lies in emerging technologies (such as AITA®) that all team members can access and that assists in getting not only the patient-specific information but also a community-level view to identify priorities. It requires us to actively use such technology and be an active member in the team. For such engagements to be successful, the role of the doctor at the clinic needs to be revisited – see Figure 1. The National Department of Health has developed guidelines on how such an approach can be integrated with the functioning of the clinic.^19^

Whilst the healthcare system reform is required, much of the innovation and direction will come from teams of healthcare practitioners learning to do things differently and seeing the healthcare system with new eyes. Focusing on building a strong team, the effective use of multiple sources of information and working towards action and improvement is the basis for such innovation and is where the doctor can have a major impact.

We need to re-equip ourselves and hone the skills and tools beyond the clinical – indeed, to understand our clinical role and decision-making in the context of the person, their world and the local system. It may be useful to group the tools and skills under a few headings:

- Getting oriented – and stay connected!
  ■ How can we know our patients (continuity of care, consultation skills, such as the three-stage assessment)
  ■ How can we get to know the community we are working in (go for a drive! Review the available data, geography, google the history and culture, political economy, stats SA)
  ■ What is the system that we are working in – resources, referrals, transport, etc.
  ■ Know your team – who is available in the immediate MDT and the extended MDT, the intersectoral players (e.g. local police), their roles, personalities, strengths, preferences) Going to visit often creates relationships that will be able to leverage cooperation!
- How to work in teams – discussion regarding clarity of roles, linkages to team members (especially not present in the clinic such as CHWs) – strengthen shared decision-making.
- Know the tools for the clinical work, functioning in the system and working in the community – be sure to know the national guidelines and protocols, forms, local arrangements, information system.
- Become competent in doing clinical governance (e.g. perinatal mortality meetings, child health priority identification programme, chart reviews, etc.). It is critical to link clinical governance at different levels to each other (clinical governance in the community, in the clinic and in the hospital). This would give a global picture of what happened with Mrs. Dlamini, for instance.
- Link clinical governance to developing a community diagnosis (linking with additional data about the community, such as infrastructure, deprivation index, etc.).
- Link the community diagnosis to how to make a difference! Quality improvement projects, community projects, link with civil society and other departments, advocacy.

It is clear that the above approach links strongly to the local context and looks different in each clinic. The approach looks different in a remote rural clinic or an inner city context with a high number of migrants. The detail of the response will vary as the available resources, geography and existing referral patterns. All of this requires agency, advocacy and agility – in any of the situations we can find ourselves.

## Conclusion

An analogy that is often used in describing the need to refocus the healthcare system is instead of focusing on mopping, turning off the tap. The doctor at the clinic is particularly well-placed to play a significant role to assist in turning off of the tap! In the kind of situations that resemble Mrs. Dlamini’s story, it is not clear whether the clinical outcome could be different. However, even at the point of returning home after the amputation, the way she would be cared for could be very different.
